# Implementing Mental Health Promotion Initiatives - Process Evaluation of the ABCs of Mental Health in Denmark

**DOI:** 10.1093/eurpub/ckac129.452

**Published:** 2022-10-25

**Authors:** L Cantarero Arevalo, C Hinrichsen, VJ Koushede, K Rich Madsen, L Nielsen, ZI Santini, C Meilstrup

**Affiliations:** 1 Danish National Institute of Public Health, University of Southern Denmark, Copenhagen, Denmark; 2 Department of Psychology, University of Copenhagen, Copenhagen, Denmark

## Abstract

Treatment and prevention alone are unlikely to make a significant difference in reducing the burden of poor mental health and mental illness. Therefore, mental health promotion (MHP) initiatives are advocated. In 2014, the ABCs of mental health (ABCs) partnership was established in Denmark; in the partnership, partner organisations, e.g., municipalities and NGOs, use a research-based framework for MHP, the ABC-framework, to develop and implement MHP initiatives. This presentation has two aims: (1) to outline the overall characteristics of these MHP initiatives; and (2) to explore local coordinator and stakeholder perceptions of the implementation processes and the impact of the MHP initiatives. Questionnaire surveys, individual interviews and group interviews were conducted during 2017-2020. The MHP initiatives were grouped according to three strategies: building MHP capacity, campaign activities to promote mental health awareness and knowledge and establishing and promoting opportunities to engage in mentally healthy activities. The ABC-framework was positively received and viewed as providing relevant knowledge for working with MHP as well as fostering intersectoral and interprofessional collaborations. However, using a bottom-up approach to develop and implement MHP initiatives can be time-consuming and resource demanding, and it requires a deliberate balancing of local adaptability and concrete guidance when engaging stakeholders and implementers. Overall, using the ABC-framework to develop and implement MHP initiatives holds great promise for advancing and promoting MHP practice.

